# Association of dietary behaviors, biochemical, and lifestyle factors with metabolic phenotypes of obesity in children and adolescents

**DOI:** 10.1186/s13098-020-00617-0

**Published:** 2020-12-07

**Authors:** Mostafa Qorbani, Pouria Khashayar, Hadith Rastad, Hanieh-Sadat Ejtahed, Ehsan Shahrestanaki, Ehsan Seif, Seyede Shahrbanoo Daniali, Masoomeh Goudarzi, Mohammad Esmaeil Motlagh, Zeinab Khodaparast, Ramin Heshmat, Roya Kelishadi

**Affiliations:** 1 Non-communicable Diseases Research Center, Alborz University of Medical Sciences, Karaj, Iran; 2 Chronic Diseases Research Center, Endocrinology and Metabolism Population Sciences Institute, Tehran University of Medical Sciences, No. 111, 19th St., North Kargar Ave, Tehran, Iran; 3 Cardiovascular Department, College of Medical, Veterinary and Life Sciences, University of Glasgow, Glasgow, UK; 4 Social Determinants of Health Research Center, Alborz University of Medical Sciences, Karaj, Iran; 5 Obesity and Eating Habits Research Center, Endocrinology and Metabolism Clinical Sciences Institute, Tehran University of Medical Sciences, Tehran, Iran; 6 Endocrinology and Metabolism Research Center, Endocrinology and Metabolism Clinical Sciences Institute, Tehran University of Medical Sciences, Tehran, Iran; 7 Dietary Supplements and Probiotic Research Center, Alborz University of Medical Sciences, Karaj, Iran; 8 Student Research Committee, Alborz University of Medical Sciences, Karaj, Iran; 9 Department of Pediatrics, Child Growth and Development Research Center, Research Institute for Primordial Prevention of Non-communicable Disease, Isfahan University of Medical Sciences, Hezar Jerib Ave, Isfahan, Iran; 10 Department of Pediatrics, Ahvaz Jundishapur University of Medical Sciences, Ahvaz, Iran; 11 Clinical Research Development Center of Kamali Hospital, Alborz University of Medical Sciences, Karaj, Iran

**Keywords:** Metabolic syndrome, Obesity, Metabolic phenotype, Children, Adolescents

## Abstract

**Background and aims:**

To examine the association of dietary behaviors, lifestyle, and biochemical factors with metabolic phenotypes of obesity among obese Iranian children and adolescents.

**Methods:**

This cross-sectional study was conducted within the framework of the fifth phase of CASPIAN study. Of 3840 students aged 7–18 years of 30 Iranian provinces, 408 subjects were diagnosed as obese; they were divided into metabolically healthy obese (MHO) and metabolically unhealthy obese (MUO) groups. Biochemical factors, anthropometric measures, dietary, and lifestyle habits were compared between groups.

**Results:**

Of the 408 obese subjects, 68 (16.7%) were the MUO; the remaining 340 (84.3%) fall in the MHO group. The MUO group had significantly higher systolic and diastolic BPs, FBS, TG, ALT, anthropometric measures, and lower HDL levels than MHO groups (all p-value < 0.05). The frequency of high birth weight (> 4000 gr) was significantly higher in the MUO group than the MHO group (p-value: 0.04). A higher percentage of individuals with breastfeeding duration ≥ 6 month was found in the MUO group (95.5% (95% CI 86.1–98.6%)) compared to MHO group (85.7% (95% CI 80.4–89.7%)) (p-value = 0.04). Among dietary and lifestyle-related behaviors, only the frequency of salty snack consumption and eating food according to the parents’ request was significantly higher in the MUO group than the MHO group (p-value < 0.05).

**Conclusion:**

Dietary habits and lifestyle factors may determine the obesity phenotypes in children and adolescents.

## Introduction

One of the most preventable causes of mortality is obesity. This is while about 100 million obese children were reported in 195 countries in 2015 worldwide, with studies for instance from the US reporting its prevalence among teenagers to be tripled from 4.6 to 20.6% in the past four decades [1].

Obesity is linked with a long list of diseases, including metabolic disorders, cardiovascular disease, non-insulin-dependent diabetes, sleep apnea, osteoarthritis, and asthma, many of which are believed to persist from childhood into adulthood [2]. As a result, childhood obesity has become the focus of attention of many public health studies, and thus, exact estimations of the incidence and severity of the disease as well as its contributors are of great importance.

Despite all this, current recommendations for the management of childhood obesity has shown limited efficacy. Many believe this is due to the fact that the essence of obesity lies in the adipose tissue and therefore body mass index is an indirect estimation of the amount of fat in the body and poor health [3]. These points out that other determinants should be used to assess obesity.

Obese individuals have heterogeneous phenotypes, each associated with various health conditions [2]. Metabolically unhealthy obesity (MUO) defines obese subjects at high risk of metabolic diseases. Metabolically healthy obesity (MHO), on the other hand, refers to obese individuals at low risk of developing cardiometabolic disorders. Compared with MUO individuals, MHOs have more abdominal fat but less visceral fat mass and fat collection in their liver and skeletal muscles [5]. Moreover, they have less ectopic fat storage and smaller adipocyte cells that serve better adipose function, resulting in higher insulin sensitivity and thus lower risk of metabolic syndrome (MetS) and type- 2 diabetes [3, 7]. Nonetheless, MHO individuals are at a higher risk of heart failure and cerebrovascular disease than normal-weight metabolically healthy individuals [6]. The majority of MHOs, also, become ‘unhealthy’ MOs at some point in their life.

As expected, there is a long list of factors that contribute to these phenotypes. This list includes but is not limited to positive family history of obesity, anthropometric profile, environmental factors such as dietary intake behaviors and lifestyle habits, and certain biochemical markers [8, 9]. A better understanding of the molecular mechanisms that promote the body fat distribution profile therefore can help differentiate and identify these phenotypes, leading to new options to fight obesity-related cardiometabolic complications.

Recent data shows a surge in the obesity rates in the developing countries, including the Middle Eastern region, mainly due to an increase in the dietary energy surpluses secondary to the dietary changes from traditional diets to fast foods and westernized foods [2, 3]. Iranian children and adolescents, similarly, are experiencing lifestyle changes, and are being affected by obesity and its complications. In the past years, many researches have studied the prevalence of childhood obesity and its risk factors among Iranians. To our knowledge, CASPIAN Study, phase V, is the first nationwide study to examine the association of dietary behaviors, biochemical, and lifestyle factors with metabolic phenotypes of obesity among obese Iranian children and adolescents [5].

## Methods

### Study population

This study was conducted within the fifth phase of a national school-based survey entitled “Childhood and Adolescence Surveillance and Prevention of Adult Non-communicable Disease” (CASPIAN-V study). CASPIAN-V was conducted on 14,400 primary and secondary school-aged students in urban and rural areas of 30 Iranian provinces in 2015. The detailed protocol of this survey has been published previously [10]. From this group, 3840 students were randomly selected for biochemical experiments. Some 408 of them were diagnosed as obese by fulfilling the criteria for general obesity defined as having age and sex-specific BMI more than 95th percentile. Obese students were categorized as MHO and MUO groups based on having at least 3 components of 5 MetS components [11]. Mets components were defined based on the Adult Treatment Panel III (ATP III) criteria modified for the pediatric age group as follows: (1) Serum TG concentration ≥ 150 mg/dl; (2) Serum HDL-C concentration ≤ 40 mg/dL; (3) Serum FBG level ≥ 100 mg/dl; (4) Abdominal obesity: waist to height ratio > 0.5; (5) Either SBP or DBP ˃ 90th percentile for age, sex, and height [12].

### Lifestyle and dietary behaviors assessment

The demographic questionnaire for pediatrics, including information about birth weight, duration of breastfeeding, eating behaviors, screen time, physical activity, and sleep duration, was completed. For evaluating eating behaviors, information about breakfast consumption (skipper, non-skipper), eating speed (slow, medium, fast), consumption at least 3 meals/day (yes/no), consumption of sugar-sweetened beverages, junk foods (including Salty snack, Sweet consumption, and Fast food), fruits, and vegetables was filled out by all the students. Information concerning eating speed was self-reported according to the question “How fast is your eating speed compared to others?” in the questionnaire. The response was based on three possible semi-quantitative categories: “slow”, “medium”, and “fast”.

Screen time (ST) was defined as the average hours per day spent watching television or using a personal computer [13]. The physical activity (PA) pattern of the students was assessed using a validated questionnaire, where questions on leisure-time physical activity, causing heavy sweating or large increases in heart rate, were asked [13].

### Anthropometric measurements and biochemical tests

The anthropometric measurements were performed by trained healthcare staff. Height was measured without shoes with 0.5 cm accuracy. Weight was measured with minimal clothes and no shoes, using a digital scale (SECA, Germany) placed on flat ground to the nearest 0.1 kg. Body mass index (BMI) was calculated as weight (kg) divided by square of height (m^2^). Waist circumference (WC) was measured at the midpoint between the lower margin of the rib cage and top of the iliac crest with a nonelastic tape to the nearest 0.1 cm [14]. Waist to height ratio (WHtR) was measured as waist (cm) divided by height (cm). Blood pressure (BP) was measured two times with a 5-minute interval in a sitting position with a standardized mercury sphygmomanometer. The mean of the two measurements was recorded. Systolic blood pressure (SBP) and diastolic blood pressure (DBP) were defined as the first and fifth Korotkoff sounds, respectively.

Fasting blood samples were taken after 12–14 h of overnight fasting. Biochemical markers including fasting blood sugar (FBS), total cholesterol (TC), low-density lipoprotein-cholesterol (LDL-C), high-density lipoprotein-cholesterol (HDL-C) and triglycerides (TG) were measured by enzymatic methods using Hitachi auto-analyzer (Tokyo, Japan) [15].

### Ethics approval and consent to participate

We obtained written informed consent from all parents and participants older than 16 years, and oral informed consent from those who were under 16 years. The study protocol was approved by the Research and Ethics Council of the Isfahan University of Medical Sciences (project number: 194049).

### Statistical analysis

All variables were checked for normality and presented as mean and 95% confidence interval (CI) for quantitative variables. Qualitative variables were presented as a percentage and 95% CI. Quantitative and Qualitative variables were compared between the MHO and MUO groups using independent samples t-test and Chi square test respectively. All analysis was adjusted using the Bonferroni post hoc test to control multiple testing problems. Multivariate logistic regression model was run for variables which were significant in univariate model, adjusting for age, sex and living area. Data analysis was done using STATA Statistical Software version 11.0 (Stata Corp LP. Package, College Station, TX, USA). P-values < 0.05 were considered as statistically significant.

## Results

Among 408 children and adolescents with obesity, defined as age and sex-specific BMI more than 95th percentile, a metabolically unhealthy and obese phenotype (MUO) was detected in 16.7% (n = 68).

Table 1 summarizes the general characteristics and biochemical factors of obese children and adolescents according to different metabolic phenotypes of obesity. The MUO group had significantly higher systolic and diastolic BPs, fasting plasma glucose, serum triglycerides, WC, z-WC, WHtR, and lower HDL levels than the “MHO group (all p-value < 0.05). There were no differences in age, sex, area of residence, and also TC or LDL levels (all p-value > 0.05).

**Table 1:** Demographic and biochemical factors of the subjects according to different metabolic phenotypes of obesity

Variables	MHO (n = 340)	MUO (n = 68)	P value
Age (year)	12.2 (11.8–12.7)	12.3 (11.7–13.0)	0.74
Sex			
Boy	59.71 (53.78–65.36)	63.24 (50.78–74.15)	0.57
Girl	40.29 (34.64–46.22)	36.76 (25.85–49.22)	
Living area			
Urban	80.29 (72.33–86.4)	82.35 (71.57–89.64)	0.67
Rural	19.71 (13.6–27.67)	17.65 (10.36–28.43)	
FBS (mg/dL)	89.87 (88.20–91.53)	95.30 (91.63–98.98)	< 0.001
HDL (mg/dL)	47.19 (46.15–48.23)	38.88 (37.15–40.60)	< 0.001
TG (mg/dL)	80.2 (75.3–85.3)	138.6 (123.2–154.0)	< 0.001
TC (mg/dL)	153.72 (149.51–157.93)	157.26 (150.15–164.37)	0.32
LDL (mg/dL)	90.47 (86.88–94.06)	90.66 (84.82–96.50)	0.94
ALT (U/L)	8.41 (7.18–9.64)	11.58 (8.78–14.39)	< 0.001
SBP (mmHg)	103.7 (102.1–105.3)	108.1 (104.5–111.6)	0.01
DBP (mmHg)	66.3 (64.8–67.8)	73.3 (70.8–75.8)	< 0.001
BMI (kg/m^2)^	26.10 (25.52–26.67)	28.11 (24.81–31.42)	0.03
BMI (z-score)	1.61 (1.48–1.73)	2.03 (1.33–2.74)	0.03
WC (cm)	78.42 (73.90–82.93)	85.88 (82.72–89.03)	< 0.001
WC (z-score)	0.96 (.58–1.33)	1.57 (1.31–1.83)	< 0.001
WHtR	0.52 (0.50–0.54)	0.56 (0.54–0.57)	< 0.001

*TC* total cholesterol, *LDL* low density lipoprotein, *WHtR* waist to height ratio; Diastolic blood pressure, *SBP* systolic blood pressure, *BMI* body mass index, *WC* waist circumference, *FBS* fasting blood sugar, *TG* triglyceride, *HDL* high density lipoprotein, *ALT* alanine transferase, *MHO* metabolically healthy obesity, *MUO* metabolically unhealthy obesity

Besides, as compared to MHO group, the MUO group had significantly higher ALT levels (mean (95% CI) 11.58 (8.78–14.39) vs. 8.41 (7.18–9.64), p-value < 0.001), BMI (28.11 (24.81–31.42) vs. 26.10 (25.52–26.67), p-value = 0.03), and z-BMI (2.03 (1.33–2.74) vs. 1.61 (1.48–1.73), p-value < 0.001).

Table 2 shows and dietary behaviors and lifestyle-related factors by metabolic phenotypes of obesity in obese children and adolescents. Frequency of high birth weight (> 4000 gr) was significantly higher in MUO group compared to MHO group (19.69% (9.37–30.01%) vs. 16.27 (9.06–23.48), p-value = 0.04).

**Table 2:** Dietary behaviors and lifestyle-related characteristics of the subject according to different metabolic phenotypes of obesity

Characteristics	MHO (n = 340)	MUO (n = 68)	P value
Birth weight (%)			
< 2500 gr	7.69 (5.75–10.21)	13.64 (7.81–22.74)	0.04
2500–4000 gr	76.04 (71.21–80.28)	66.67 (55.36–76.33)	
> 4000 gr	16.27 (9.06–23.48)	19.69 (9.37–30.01)	
Breastfeeding duration (%)			
< 6 month	14.24 (10.21–19.52)	4.478 (1.34–13.85)	0.04
≥ 6 month	85.76 (80.48–89.79)	95.52 (86.15–98.65)	
Breakfast consumption (%)			
Non skipper	86.57 (81.63–90.33)	81.82 (68.72–90.21)	0.18
Skipper	13.43 (9.66–18.37)	18.18 (9.78–31.28)	
Eating speed of student (%)			
Slow	30.27 (24.87–36.28)	23.53 (14.17–36.45)	0.49
Average	51.63 (45.76–57.46)	54.41 (41.33–66.91)	
Fast	18.1 (14.55–22.29)	22.06 (13.34, 34.23)	
When eating food? (%)			
By child request	77.48 (69.25–84.01)	65.67 (53.98–75.73)	0.04
By parents request	22.52 (15.99–30.75)	34.33 (24.27–46.02)	
Physical activity (%)			
High	43.28 (38.61–48.08)	39.71 (26.7–54.36)	0.62
Low	56.72 (51.92–61.39)	60.29 (45.64–73.3)	
Sweetened beverage (%)			
Non daily	88.1 (83.35–91.63)	85.07 (75.49–91.34)	0.50
Daily	11.9 (8.375, 16.65)	14.93 (8.659–24.51)	
Fresh fruit consumption (%)			
Daily	60.26 (52.93–67.17)	62.71 (52.2–72.14)	0.68
Non daily	39.74 (32.83–47.07)	37.29 (27.86–47.8)	
Vegetable consumption (%)			
Daily	31.85 (26.29–37.97)	33.82 (20.48–50.35)	0.80
Non daily	68.15 (62.03–73.71)	66.18 (49.65–79.52)	
Milk consumption (%)			
Daily	36.76 (31.22–42.68)	42.65 (31.68–54.39)	0.38
Non daily	63.24 (57.32–68.78)	57.35 (45.61–68.32)	
Fast food consumption (%)			
Non daily	89.71 (85.56–92.76)	83.58 (69.32–91.98)	0.21
Daily	10.29 (7.24–14.44)	16.42 (8.018–30.68)	
Sweet consumption (%)			
Non daily	81.18 (76.81–84.88)	76.47 (67.89–83.32)	0.30
Daily	18.82 (15.12–23.19)	23.53 (16.68–32.11)	
Salty snack consumption (%)			
Non daily	95.55 (92.21–97.5)	85.29 (70.7–93.31)	0.008
Daily	4.451 (2.504–7.79)	14.71 (6.693–29.3)	
Watching TV (hours/day)	1.97 (1.86–2.09)	1.94 (1.69–2.18)	0.78
Working PC (hours/day)	0.59 (0.48–0.70)	0.52 (0.28–0.75)	0.54
Screen time (hours/day)	1.28 (1.19–1.37)	1.23 (1.06–1.39)	0.53
Sleep duration (hours/day)	8.55 (8.40–8.70)	8.40 (8.13–8.67)	0.38

The MUO group had a higher percentage of individuals with breastfeeding duration ≥ 6 month (% (95% CI) 95.52% (86.15–98.65%)) than the MHO group (85.76% (80.48–89.79%)) (p-value: 0.04).

In addition, daily salty snack consumption was significantly more frequent among individuals in the MUO group (14.71% (6.693–29.3)) compared to the MHO group (4.451% (2.504–7.79%)) (p-value: 0.008). The frequency of eating food according to the parents’ request was higher in the MUO group in comparison with the MHO group (p-value: 0.04). Results of multivariate logistic regression model showed significant association between metabolic phenotypes of obesity and salty snack consumption (OR: 3.83, 95% CI 1.63–9.03), breastfeeding duration (OR: 3.55, 95% CI 1.07-11.84), and when eating food (OR: 1.77, 95% CI 1.001–3.12), after adjusting sex, age and living area.

Other dietary behaviors (including breakfast consumption, sweetened beverage consumption, fresh fruit consumption, vegetable consumption, milk consumption, fast food consumption, and sweet consumption), eating speed, and lifestyle behaviors (including physical activity, watching TV, working with computer, screen time, and sleep duration) in the MUO group were not statistically significant compared to those in MHO group (all p-value > 0.05).

## Discussion

Our study shows a significant difference in the profile of obesity-related biochemical markers and some lifestyle habits between the MUO and MHO adolescents. Among dietary behaviors, only daily consumption of salty snack was more frequent in the MUO group.

MHO group consisted 83% of our obese population. This is while many studies have suggested that about 10–40% of the obese adult population do not experience any metabolic complications [3]. The fact that a large number of MHO children become MUO some time in their childhood or adulthood can explain the high proportion of MHOs to MUOs reported in this study. Comparing the results of this study with that in literature is challenging as there is no unique definition of MHO/MUO and this may explain the discrepancies noted in these comparisons.

Demographic factors are considered as an important predictor of obesity phenotypes, with some studies reporting a higher incidence of MHO among girls and younger adolescents [8, 10]. Similarly, more healthy obese individuals are reported to be living in rural areas, mainly because of their healthier lifestyle and diet [16]. In our study, however, no such a difference was noted between the age or sex or the living area of the two phenotypes. As expected, the anthropometric profile is another important determinant of the obesity phenotypes. In corroboration with previous studies, our results revealed a higher BMI, WC, and WHtR among MUO adolescents compared with MHO ones [8, 9].

Recent advancement in medicine has shifted the focus towards precision medicine, which refers to the “tailoring of medical treatment to the individual characteristics of each patient” and the “ability to classify individuals into subpopulations that differ in their susceptibility to a particular disease, in the biology or prognosis of those diseases they may develop, or in their response to a specific treatment” [12]. This approach believes treatment is more effective when the variability in genes, environments, and lifestyles are taken into account.

As mentioned earlier, the current approach to obesity, especially childhood obesity, has not been successful and behavioral phenotyping can thus help provide a more personalized solution to this problem. Behavioral phenotyping refers to distinct personal behaviors that result from the interaction of genes and environment [13]. They can be used to predict certain response and adopt corresponding interventions to prevent from or treat them. In this study, this was assessed through reviewing the dietary behavior or lifestyle habits of the subjects.

The dietary pattern (the time of having the meals, the speed of eating, and the type of foods) determine the cardiometabolic health status in adolescents, especially boys [8]. Skipping breakfast or having dinner late is reported as one of the main known contributors to obesity and cardiovascular diseases in adolescents [8, 14]. This is while our study only found the time of having dinner, neither the eating speed nor skipping/not skipping breakfast, linked with the obesity phenotypes. On the contrary to the previous studies, we also failed to show any correlation between following a healthy diet rich in fresh fruit, vegetables, and milk and the obesity phenotypes. Having the habit of eating salty snacks was the only routine significantly correlated with unhealthy obesity phenotypes. Our contradictory findings could be due to the fact that all of the studied participants were obese, and the studied dietary factors are known contributors to obesity in general. It is possible that different results would have been obtained if these habits were compared with that of normal-weight adolescents.

There is no consensus about the association of certain lifestyle habits with the metabolic phenotypes of obesity. Several studies have shown a higher incidence of obesity among children and adolescents with high screen time (TV and PC) [7, 8]. This is while others have not found any link between following a sedentary lifestyle and these phenotypes [15]. In corroboration with the latter, our study did not find any significant relation between screen time and the obesity phenotypes. The fact that screen time alone is a determinant of a specific type of sedentary lifestyle and the other types should also be studied to have a better idea of the differences in the activity profile of these two groups.

Poor sleeping quality (having trouble falling asleep, feeling unrested or being sleepy during the day) and low sleep duration are reported to be associated with MUO, mainly among boys [8, 9]. Our study did not find any correlation between the duration of sleep and obesity phenotypes. Again, the fact that both groups are obese and having a sedentary lifestyle and poor sleep are known risk factors for obesity could have resulted in these findings.

Several biochemical markers are among other determinants of obesity phenotypes, with many studies reporting lower levels of fasting triglycerides and fasting glucose but elevated levels of high-density lipoprotein (HDL) among MHOs compared with MUOs [9]. On the contrary to these studies, the MUOs in our survey were reported to have higher TG and HDL levels but not TC and LDL levels [8, 16]. On the other hand, in line with previous studies, higher ALT levels were reported to be responsible for the increased risk of cardiometabolic diseases among MUO boys in our study [8, 17].

Obese children are reported to have higher blood pressure values compared with their normal weighted counterparts, which is believed to make them susceptible to many diseases, mainly cardiometabolic disorders [16]. Similar to previous studies, our results showed higher BP values, both SBP and DBP, in MUO adolescents.

Apart from the adolescent behavior and habits, their weight at birth and the duration of breastfeeding are also reported to affect the obesity phenotypes. This is inconsistent with previous studies that had considered lower birth weight and longer breastfeeding duration as protective factors against obesity [8, 18, 19].

The present study is one of the largest cohorts on obese adolescents from different parts of the country examining various determinants of metabolic phenotypes. As a result, its finding could be generalized to the Iranian adolescents. Among various factors linked with obesity and its phenotypes, the present study has assessed the influence of most important risk factors of obesity, such as dietary factors, lifestyle habits, and certain biochemical factors (lipid and liver function). As for the anthropometric profile, however, we only used BMI as a measurement of obesity, while this value cannot distinguish between fat and lean tissue [11]. This issue could be pointed as one of the limitations of the present study. Moreover, there is no definite definition for MHO/MUO, resulting in different studies applying various criteria for this matter. It is possible that the application of other definitions to distinguish these groups could have resulted in different results. In this regard, future cohort studies are needed to define the best MHO/MUO definition for the Iranian childhood population through studying the metabolic complications. Furthermore, as mentioned earlier this study was only conducted on obese adolescents and the fact that all of the studied variables are known risk factors of obesity in general could explain our findings. Considering the low sample size of MUO group and sparse of data, confidence intervals of multivariate logistic regression are wide. Therefore, the findings should be interpreted cautiously. Further studies with large sample size are needed to investigate the effect of diet/lifestyle factors as well as other factors such as the quality of sleep (insomnia), uric acid levels, family history of metabolic diseases, race or second-hand smoke as the predictors of MUO.

## Conclusion

Behavioral phenotyping through defining obesity phenotypes helps identify the variation in children’s dietary behaviors and lifestyle factors, determine those at risk and adopt the personalized intervention based on the individual’s predisposition to prevent obesity later in the child’s life. However, additional research is needed to improve risk stratification for these phenotypes. The results of such studies could in near future result in targeted screening along with cost-effective detection and personalized treatment of children at higher risk for cardiometabolic disorders.

## Data Availability

The datasets used and/or analyzed during the current study are available from the corresponding author on reasonable request.

## Abbreviations

ALT: Alanine aminotransferase
ATP III: Adult treatment panel III
BMI: Body mass index
BP: Blood pressure
CASPIAN: “Childhood and Adolescence Surveillance and Prevention of Adult Non-communicable Disease”
CI: Confidence interval
DBP: Diastolic blood pressure
FBS: Fasting blood sugar
HDL-C: High-density lipoprotein-cholesterol
LDL-C: Low-density lipoprotein-cholesterol
MetS: Metabolic syndrome
MHO: Metabolically healthy obesity
MUO: Metabolically unhealthy obesity
PA: Physical Activity
SBP: Systolic blood pressure
ST: Screen time
TC: Total cholesterol
TG: Triglycerides
WC: Waist circumference
WHtR: Waist to height ratio
NCD: Non-communicable diseases
